# An exon skipping screen identifies antitumor drugs that are potent modulators of pre-mRNA splicing, suggesting new therapeutic applications

**DOI:** 10.1371/journal.pone.0233672

**Published:** 2020-05-29

**Authors:** Yihui Shi, Walter Bray, Alexander J. Smith, Wei Zhou, Joy Calaoagan, Chandraiah Lagisetti, Lidia Sambucetti, Phillip Crews, R. Scott Lokey, Thomas R. Webb

**Affiliations:** 1 Bioscience Division, SRI International, Menlo Park, CA, United States of America; 2 Department of Chemistry and Biochemistry, University of California, Santa Cruz, CA, United States of America; University of Iowa, UNITED STATES

## Abstract

Agents that modulate pre-mRNA splicing are of interest in multiple therapeutic areas, including cancer. We report our recent screening results with the application of a cell-based Triple Exon Skipping Luciferase Reporter (TESLR) using a library that is composed of FDA approved drugs, clinical compounds, and mechanistically characterized tool compounds. Confirmatory assays showed that three clinical antitumor therapeutic candidates (milciclib, PF-3758309 and PF-562271) are potent splicing modulators and that these drugs are, in fact, nanomolar inhibitors of multiple kinases involved in the regulation the spliceosome. We also report the identification of new SF3B1 antagonists (sudemycinol C and E) and show that these antagonists can be used to develop a displacement assay for SF3B1 small molecule ligands. These results further support the broad potential for the development of agents that target the spliceosome for the treatment of cancer and other diseases, as well as new avenues for the discovery of new chemotherapeutic agents for a range of diseases.

## Introduction

The use of targeted high-throughput screening (HTS) of recently available compound libraries composed of drugs, clinical compounds and advanced tool compounds offers the biomedical research community the opportunity to elucidate the mechanism of action (MOA), on-target specificity and potential for clinical repositioning of specific drugs, while at the same time developing a refined drug candidate profile for researchers in specific areas of drug discovery and drug development. The spliceosome is accountable for the post-transcriptional processing of pre-mRNA in the cells of metazoans by catalyzing the regulated exclusion of intervening sequences (introns) and the ligation of coding regions (exons) to produce mature mRNAs, and has recently emerged as a novel target in several therapeutic areas.[1] Small molecules that affect AS have been of interest for numerous therapeutic applications since they impact cellular function by modifying the abundance of different splicing isoforms that play a role in numerous disease states.[2]

Given the important role that the spliceosome plays in the determination of cellular and organismal phenotypes it is not surprising that the function of the spliceosome is aberrant in most tumors.[3] Indeed, numerous genes are subject to splicing events that can be either oncogenic or serve to limit potential tumorigenesis, examples of this include BCL-X, VEGF-A, FAS, PKM or MDM2.[4] Additionally, numerous recurrent mutations occur in spliceosome regulatory components (including SF3B1, SRSF2, U2AF1 and others) in the myelodysplastic syndromes and other cancers.[5] These mutations result in a ‘change in function’ of the mutant spliceosome and a consequential change in the AS profile in the cells expressing these mutant proteins.[6–8]

In parallel to these recent discoveries, there has been a proportional upsurge in interest in the potential application of several recently discovered small molecule modulators of pre-mRNA splicing to cancer chemotherapy.[9–11] This effort has resulted in Phase I clinical studies and advanced pre-clinical development, for a series of ligands of the SF3B1 spliceosomal protein. These innovative drugs include a derivative of the natural product pladienolide (E7107),[12] a synthetic analog of pladienolide[13, 14] (H3B-8800),[15] and sudemycin D6 (SD6)[16] a simplified synthetic analog of a natural product (FR-901,464).[17] SD6 is currently actively advancing through the ‘investigational new drug’ (IND) development process. Although the natural products which inspired these drugs were initially described as “splicing inhibitors”,[12, 17] we now know that SF3B1 targeted agents act as potent modulators of AS through a change in 3’ splice-site fidelity.[18–20] Tumor cells exposed to the splicing modulatory natural products (and analogs) display a profound change in AS,[19, 20] which shows similarities to the pharmacology that has been observed with kinase inhibitors that interfere with the regulatory phosphorylation of splicing factors.[10]

Although the full range of molecular mechanisms responsible for the tumor selective toxicity of these agents remains to be fully elucidated, several mechanism types have been delineated. An early mechanism class to to be recognized is the sensitivity of tumor cells bearing spliceosomal mutations, for example chronic lymphocytic leukemia (CLL) cells bearing SF3B1 mutations, [21] and myelodysplastic syndrome (MDS) cells carrying U2AF1 mutations.[22] Additionally, it was found that tumors driven by MYC[23] or KRAS[24] are also sensitized to this class of drugs. More recently proposals have appeared for two additional general mechanisms that may account for the observed selective action of SF3B1 targeted agents in certain cancers, the first proposes that ~11% of all cancers have a partial copy of wild-type SF3B1 protein, which renders these tumors sensitive to SF3B1 targeted drugs;[25] another recent publication presents data which is consistent with the idea that certain tumors driven by BCL2A1, BCL2L2 and MCL1 are especially susceptible to SF3B1 targeted agents.[26] It is certainly possible that multiple mechanisms can account for the selective tumor toxicity that has been observed with these agents, which supports the concept that these agents have good potential for broad application in cancer chemotherapy.[9]

Given these new insights into the relationships between carcinogenesis and spliceosome function we initiated a project aimed at the discovery of additional small molecules that target the spliceosome. This has been facilitated by our Triple-Exon Skipping Luciferase Reporter (TESLR) cell-based HTS assay,[27] which reports on a particular type of triple-exon skipping event in MDM2 pre-mRNA that we first observed in tumor cells treated with sudemycin analogs.[19] We applied this HTS assay to the unbiased screen of a collection of all FDA approved drugs, bioactive compound collections, and compounds in Phase I through Phase III clinical trials, in order to build on our previously reported pilot screening results with this assay, which identified two known cyclin-dependent kinases (CDK) inhibitors that were found to also inhibit several members of the cdc-like kinase (CLK) family. The hits from this pilot screen were then developed into SRI-30125, a selective inhibitor of CLKs 1, 2, and 4.[28] Importantly this work also showed that aminopurvalanol,[29] a commonly used tool compound that is often considered a “selective CDK2 inhibitor” is actually a multi-kinase inhibitor and a potent modulator of AS, which we showed acts through the inhibition of CLKs 1, 2, and 4.[28]

**Fig 1:**
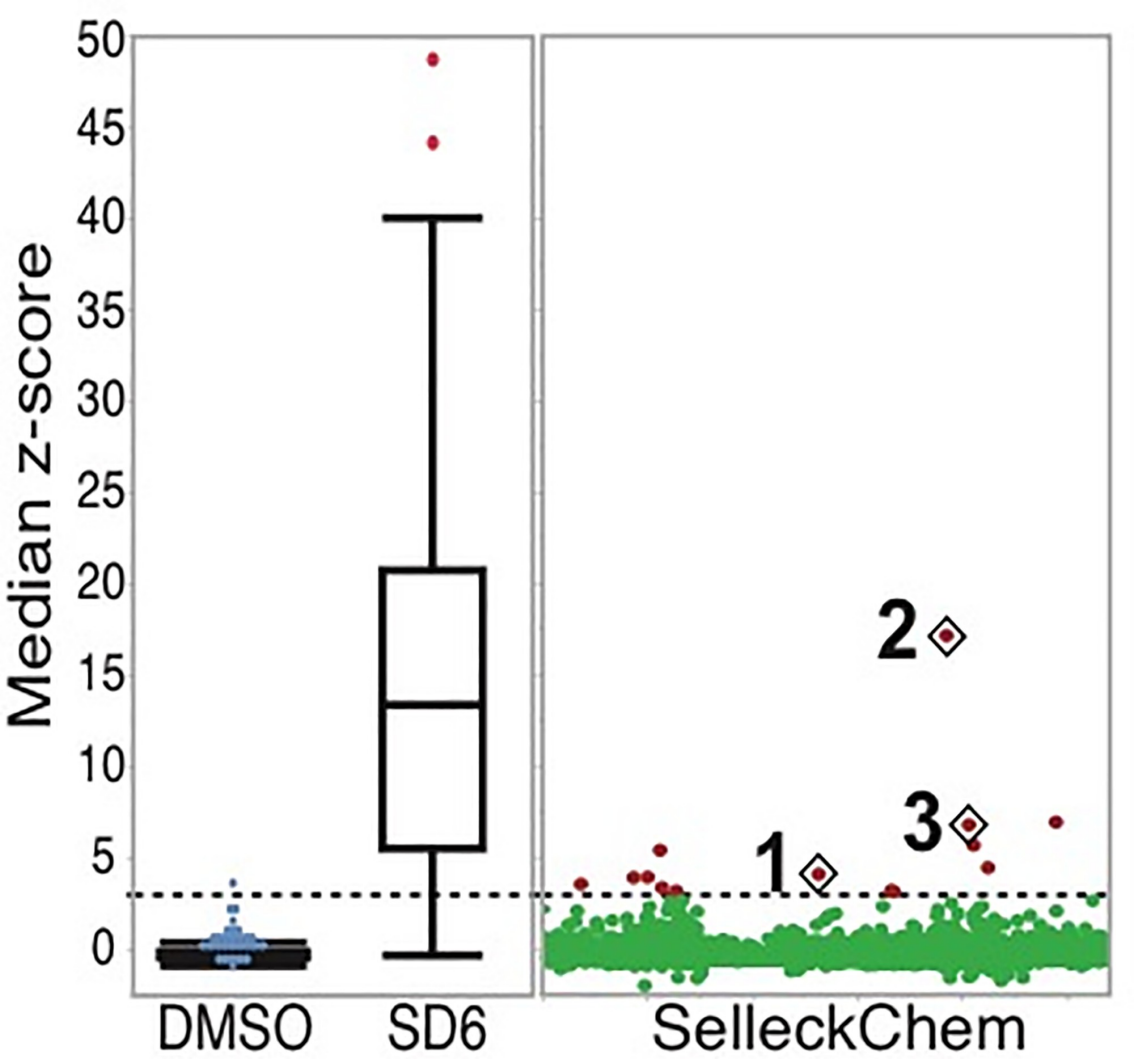
HTS screen. Screening results showing median Z-scores from three replicates, with the combined negative (DMSO) and positive (SD6) controls shown on the left, and the complete dataset from the SelleckChem library on the right. Shown in red are the compounds that were selected for follow-up dose-response studies based on their median Z-scores (>3), and diamonds mark the three compounds that showed a dose-response upon retesting.

This paper describes the results from our clinical compound focused HTS screen (see Fig 1), which led to the identification of three potent post-Phase I splicing modulatory drugs (**1**, **2**, and **3**: see Table 1),[30–32] that have not been previously recognized as splicing modulators. These investigational drugs were developed to target oncogenic kinases and were not previously reported to inhibit splicing regulatory kinases. All of these confirmed hits have been previously reported to display potent *in vivo* antitumor activity, which was proposed to be due to their activity to other kinases (CDK2, PAK4 or FAK, respectively), however we now report that they are also nanomolar inhibitors of subsets of the multiple kinases involved in the regulation of spliceosome activity. These observations also align with the previous report that another clinical antitumor agent CX-4945 (Silmitasertib) inhibits splicing regulatory kinases and modulates alternative splicing.[33] CX-4945 was developed as a CK2 inhibitor but was later recognized to be a modulator of alternative splicing following the initiation of clinical studies.[33] As discussed below, we propose that the splicing modulatory activity may positively contribute to the antitumor activity of all of these clinical drug candidates.

**Table 1:** Substrates and conditions for biochemical IC_50_ determinations.

Enzyme^a^	Substrate (concentration)	ATP (μM)	%CV^g^
AKT3	GRPRTSSFAEGKK (30μM)	45	3
CDK2/cyclinA (h)	histone H1 (0.1mg/mL)	45	6
ClKl(h)^b^	ERMRPRKRQGSVRRRV (200μM)	10	2
ClK2(h)	YRRAAVPPSPSLSRHSSPHQS(p) EDEEE (20μM)	10	4
ClK3(h)^c^	ERMRPRKRQGSVRRRV (250μM)	90	4
ClK4(h)	YRRAAVPPSPSLSRHSSPHQS(p)EDEEE (200μM)	10	3
DYRKIA(h)	RRRFRPASPLRGPPK (50μM)	45	7
DYRKIB (h)	RRRFRPASPLRGPPK (50μM)	45	4
DYRK2 (h)	casein (2mg/ml)	15	3
SRPKl(h)	RSRSRSRSRSRSRSR (300μM)	10	10
SRPK2 (h)	RSRSRSRSRSRSRSR (300μM)	10	4
SRPK3 (h)	ERMRPRKRQGSVRRRV (50μM)	15	7
CK2 (h)^d^	RRRDDDSDDD (165μM)	10	5
CK2α2 (h)^d^	RRRDDDSDDD (330μM)	10	3
GSK3α(h)	YRRAAVPPSPSLSRHSSPHQS(p) EDEEE (20μM)	10	6
GSK3β(h)	YRRAAVPPSPSLSRHSSPHQS(p) EDEEE (20μM)	15	6
MAPK2 (h)^e^	myelin basic protein (0.33 mg/mL)	155	5
PYK2 (h)	poly(Glu, Tyr) 4:1(0.1 mg/mL)	90	5
PAK4 (h)	myelin basic protein (0.8 mg/mL)	10	7
FAK (h)^f^	EEEEYEEEEEEYYllEEEEEEYEEEEEEYYEEEEEEKKKK (100 μM)	70	5
NEK2 (h)	myelin basic protein (0.33 mg/mL)	120	4
Pim-1 (h)	KKRNRTLTV (100μM)	90	4
Pim-2 (h)	RSRHSSYPAGT (300μM)	15	10

^a^ The standard enzyme reaction buffer contains mM MOPS pH 7.0, 10 mM Mg Acetate, 0.2 mM EDTA. The additional components of any specific enzyme are described from b-k.

^b^ The enzyme reaction buffer for CLK1(h) also contains 1 mM sodium orthovanadate, 5 mM sodium 6-glycerophosphate.

^c^ The enzyme reaction buffer for CLK3(h) also contains 30 mM NaCl.

^d^ The enzyme reaction buffer for CK2 (h) and CK2α2 (h) also contain 20 mM HEPES, 0.15 M NaCl, 0.1 M EDTA, 5 mM DTT, 0.1% Triton X-100, 50% Glycerol.

^e^ The enzyme reaction buffer for MAPK1 (h) and MAPK2 (h) also contain 50 mM TRIS, 0.1 mM EGTA, 0.1 mM Na_3_VO_4_, 0.1% 6-mercaptoethanol, 1 mg/mL BSA.

^f^ The enzyme reaction buffer for FAK (h) also contains 15 mM MOPS, 0.75 mM EDTA, 0.0075% Brij-35, 3.75% Glycerol, 150 mM NaCl, 0.1% 6-mercaptoethanol, 1 mg/mL BSA.

^g^ Percent CV is of the assay is calculated from the CV of four control compound repeats across different experiments.

## Materials and methods

### High throughput screening methods

SK-Mel-2-MDM2-Luc cells were cultured in MEM supplemented with 10% fetal bovine serum, 1 mM pyruvate, and 0.1 mg/mL of the antibiotic G418; the cells were grown in a 37°C incubator at 5% CO_2_.[28] To perform the TESLR high-throughput screen, white 384 plates were seeded with 20 μL per well of growth medium without G418, at a density of 5,000 cells per well using a Multidrop peristaltic pump (Thermo Scientific). After 18 h at 37°C in an incubator in 5% CO_2_, cells were treated with 100 nL of compound using a pin tool attachment for a Janus MDT automated workstation (Perkin Elmer). SD6 at 5 μM was used as positive control for splicing modulation. After 5 h incubation at 37°C with 5% CO2, an equal volume of OneGlow XL (Promega) was added using a Matrix WellMate dispenser (Thermo Scientific). Luminescence readings were taken immediately using an Envision multilabel reader (PerkinElmer). We screened 2035 compounds (seven 384-well plates) from SelleckChem at 10 μM in triplicate. The first 2 columns were reserved for the SD6 positive control, while the last two columns contained DMSO as a negative control. Z-scores were calculated from the DMSO-treated negative controls using the following equation: [X–μ(DMSO)]/σ(DMSO), where X = the luminescence intensity for a given well, and μ(DMSO) and σ(DMSO) are the average and standard deviation of the DMSO (calculated for each plate separately). Hits were determined as those wells having a median Z-score greater than 3. There were 12 hits that scored as positives by these criteria in both replicates, and these were repurchased, their purities confirmed by LCMS, and submitted to dose-response testing for confirmation of splicing modulation activity and to determine potency. All other experimental details are included in the Supporting Information section.

### TESLR dose response

SK-MEL-2/MDM2-Luc stable cells were cultured in MEM medium with Earle's salts and L-glutamine containing 1 mM sodium pyruvate, 10% FCS and 10 mM Hepes and plated at a density of 10,000/well in 96-well plates and incubated overnight at 37°C in 5% CO_2_. The following day, cells were treated with serial dilutions of compounds for 4 hrs, ONE-Glo reagents (Promega) were added to measure the luciferase activity on an EnVision plate reader. SD6 and 0.5% DMSO were used as positive and negative controls, respectively. Relative luminescent units were plotted against corresponding drug concentrations and fitted with a standard four parameter sigmoidal curve with GraphPad Prism.

### Biochemical kinase inhibition assays

Enzymatic biochemical activities were evaluated in radiometric protein kinase assays (Eurofins, Dundee, Scotland). Kinases are incubated with buffer, substrate and [γ-^33^P]-ATP (specific activity approx. 500 cpm/pmol, concentration as required). The reaction is initiated by the addition of the MgATP mix. After incubation for 40 minutes at room temperature, the reaction is stopped by the addition of 3% phosphoric acid solution. 10 μL of the reaction is then spotted onto a P30 filtermat and washed three times for 5 minutes in 75 mM phosphoric acid and once in methanol prior to drying and scintillation counting. All compounds are prepared to 50x final assay concentration in 100% DMSO. Positive control wells contain all components of the reaction with 2% DMSO. Blank wells contain all components of the reaction, with a reference inhibitor replacing the compound of interest. Each kinase is assigned a standard assay concentration of ATP within 20 μM of its apparent K_m_. Compounds were screened at 1 μM in duplicate or tested at 10 concentrations at half log dilution starting from 10 μM in singlicate. Dose response curves were fitted with four parameter logistic curve to obtain IC_50_ values. The average coefficient of variation (CV) of the conducted assay.

Percent inhibition was calculated by comparing to the positive control wells that contain all components of the reaction and 2% DMSO instead of compound (0% inhibition), as well as the blank wells that contain all components of the reaction, with a reference inhibitor (100% inhibition). Staurosporine is used as reference for all tested enzymes except for SRPK3 (reference inhibitor: K-252a. Nocardiopsis sp), NEK2 (reference inhibitor: 30% phosphoric acid), and CK2, CK2α2, MAPK1, MAPK2 (reference inhibitor: PKR Inhibitor). See Table 1 below for additional details.

### Confirmation of splicing changes with RT-PCR

RH-18 cells were cultured in RPMI 1640 medium containing 10% FCS. Cells are all maintained in humidified incubator with 5% CO2 at 37°C. Rh18 cells were exposed to 0.5% DMSO or SD6 10 μM, Cpd1 10 μM, Cpd2 5 μM or Cpd3 10 μM for 4 h. Total RNA was extracted and converted to cDNA. PCR was performed using NEB Q5 Master Mix and specific primers listed below (Table 2) according to the manufacturer’s instruction and standard PCR protocols: 50 ng cDNA and 30 μl of the final reaction volume was used. PCR products were subjected to 3% agarose gel electrophoresis.

**Table 2:** List of primers used in the RT-PCR.

primer	sequence
MDM2 Forward	CTGGGGAGTCTTGAGGGACC
MDM2 Reverse	CAGGTTGTCTAAATTCCTAG
UBQ Forward	ACCTGACCAGCAGCGTCTGATATT
UBQ Reserve	TCGCAGTTGTATTTCTGGGCAAGC
RBM39 Forward	GAAGCAATGCTTGAGGCTCC
RBM39 Reverse	GCTCAAAGATCCCACGAAGC

### Western blot methods

HCT116 cells were seeded at 3 million cells per 100 mm culture plate. The next day cells were treated with 5 μM or 10 μM of each compound for 4h and 8h. For harvesting, cells were washed 2X with ice cold PBS and then resuspended in 400 μl of nuclear isolation lysis buffer (150 mM NaCl, 10 mM Tris-HCl pH 7.4, 1 mM EDTA pH8, 0.5% NP-40, 1X Halt Protease and Phosphatase Inhibitor). Cells were scraped and transferred to a microfuge tube and spun at 2800 rpm for 5 minutes, 4C. The supernatant was removed and the nuclear pellet was resuspended in a nuclear extraction buffer (400 mM NaCl, 20 mM Hepes pH 7.9, 1 mM EDTA pH 8, 1 mM DTT, 1X Halt Protease and Phosphatase Inhibitor). The pellet was snap frozen on dry ice and then thawed and spun at 12K rpm for 5 min, 4C. The supernatant was collected (nuclear extract) and protein concentrations determined using BCA Protein Assay Kit. Samples were run alongside molecular weight markers (Invitrogen SeeBlue™ Plus2 #LC5925) on 4–12% Bis-Tris protein gels (Invitrogen# NP0323BOX) using MOPS SDS running buffer (Invitrogen# NP0001). The proteins were transferred to PVDF membrane using a wet transfer system. The membranes were incubated for 30 minutes at room temperature in Odyssey blocking buffer (Li-Cor 927–40000), then incubated overnight at 4°C with primary antibody in blocking buffer containing 0.2% Tween-20 and 1h at 4°C with secondary antibody in blocking buffer containing 0.2% Tween-20 and 0.02% SDS. After the secondary antibody incubation, membranes were washed 4X with PBS containing 0.1% Tween-20 and 1X with PBS before imaging using the Li-Cor Odyssey imaging system. The primary antibodies used were as follows: SR Monoclonal Antibody (16H3) (Thermo Fisher #16H3E8), Phospho-SF3B1 (Thr313) (D8D8V) (Cell Signaling #25009), Anti-SF3B1 antibody [EPR11987(B)] (Abcam#170854), Anti-phosphoepitope SR protein (clone 1H4) (Millipore# MABE50). The secondary antibodies used are as follows: IRDye® 800CW Goat-anti-Mouse (Li-Cor 925–32210) and IRDye® 680RD Goat-anti-Rabbit (Li-Cor 926–68071).

### Antagonist screen

SK-MEL-2/Luc-MDM2 stable cells were plated at a density of 20,000 cells/well in 96-well plates and incubated overnight at 37°C in 5% CO_2_.[27, 28] The following day, cells were treated with 14 analogs using serial dilution for one hour. The list of compounds screened as potential TESLR antagonists are show in SI file. After one-hour pre-treatment, 4 μM of SD6 were added to the pre-treated cells for another 4 hours. Either 0.5% DMSO or serial dilutions of SD6 were used to treat cells for 4 hours as controls. At the end of incubation time, ONE-Glo™ EX reagents (Promega) were added to measure the luciferase activity. The graph shows the luc intensity value of DMSO or 4 μM of SD6 that were the average of the reading of cells treated with DMSO or 4 μM of SD6 for 4 hours only. These compounds showed no detectable or weak cytotoxic effects at 10 μM, and so were deemed ‘inactive’ according to our published standard criteria (72 h cytotox EC_50_ > 1 μM).[16]

### Chemistry

Unless otherwise noted, all commercial reagents were obtained from commercially available sources and used without purification. Flash column chromatography was performed on a Biotage SP-1 chromatography system. TLC plates were visualized by exposure to ultraviolet light (254 nm). ^1^H and ^13^C spectra were recorded using 400 MHz, and 300 MHz, respectively, using CDCl_3_, CD_3_OD, or DMSO-*d*_6_ as a solvent. The chemical shifts are reported in parts per million (ppm) relative to residual solvent (for chloroform, *δ* 7.24 ppm for ^1^H NMR and *δ* 77.02 ppm for ^13^C NMR. For DMSO, *δ* 2.47 ppm for ^1^H NMR. For CD_3_OD, *δ* 49.00 ppm for ^13^C NMR). Coupling constants are reported in hertz (Hz). The following abbreviations are used to designate the multiplicities: s = singlet, d = doublet, t = triplet, q = quartet, m = multiplet. Mass spectra with electrospray ionization (ESI) were recorded on LCQ Fleet Ion Trap Mass Spectrometer (Thermo Scientific) coupled to the Finnigan Surveyor Plus HPLC System (Thermo Scientific). High-resolution mass spectra were recorded on LTQ-Orbitrap XL (Thermo Scientific) using static nanoelectrospray ionization in positive-ion profile mode at a nominal resolution setting of 100,000. Approximately 50 scans were averaged for each sample and the resulting Fourier-transformed frequency-domain spectrum was mass-assigned with calibration constants from an external calibration mixture. Experimental masses and isotope distributions were compared to theoretical values. All compounds reported are of at least 95% purity, as judged by HPLC (Waters XBridge C18, 250 mm X 4.6 mm ID, 5 μm column; 10 μL injection; 10–100% MeCN/H_2_O + 0.1% TFA gradient over 15 min; 1 mL/min flow; ESI; positive ion mode; UV detection at a wavelength of 310 or 340 nm).

## Results

### An unbiased screen of drugs and drug candidates identifies three investigation antitumor drugs that potently induce the same exon skipping event as the SF3B1 targeted antitumor drug SD6

**Fig 2:**
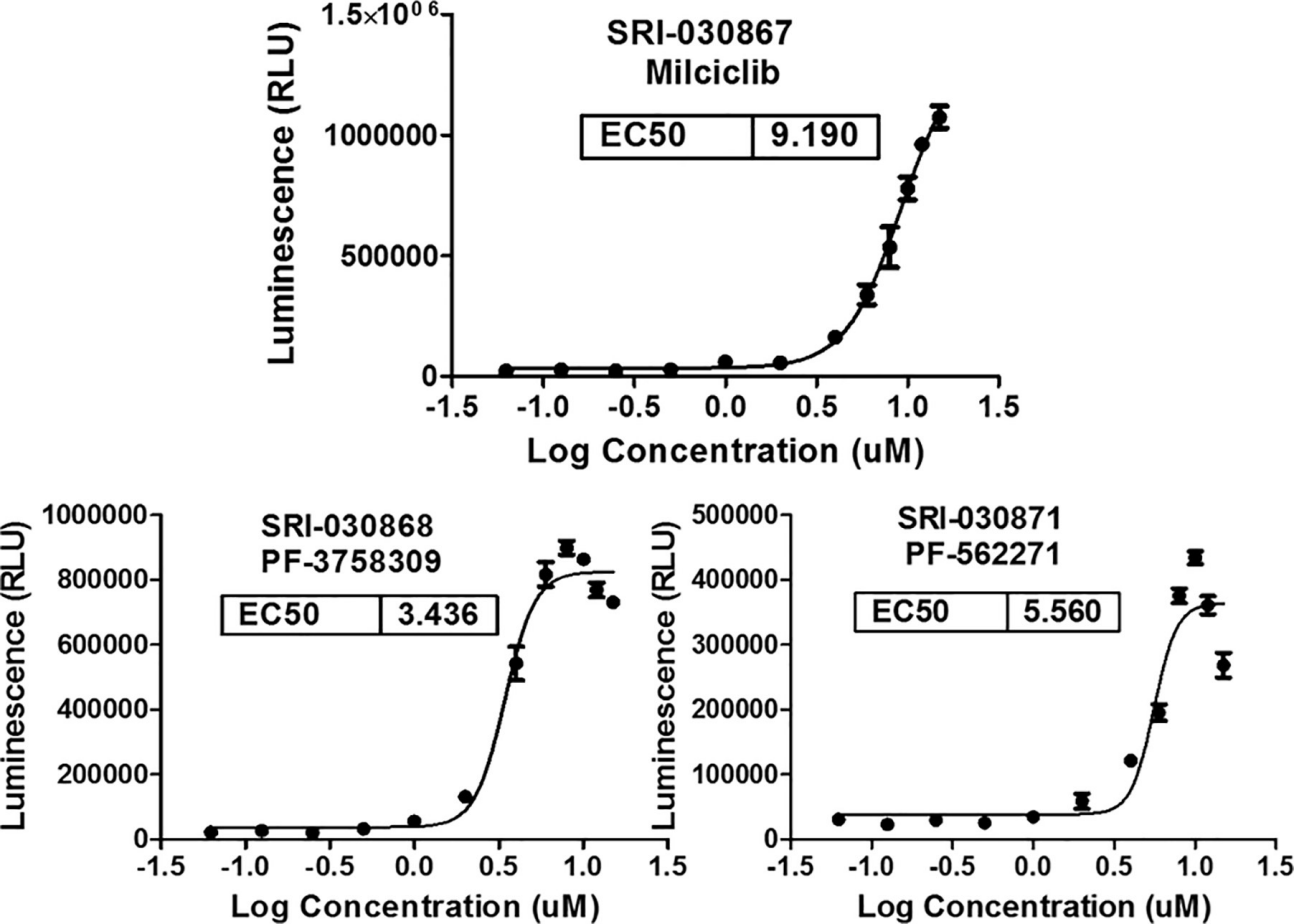
Dose response curves of three hits that were confirmed active. The TESLR assay in SK-MEL-2/Luc-MDM2 cells with 4 hour treatment. SK-MEL-2/Luc-MDM2 stable cells were plated at a density of 20,000 cells/well in 96-well plates and incubated overnight at 37°C in 5% CO_2_. The following day, cells were treated with either 0.5% DMSO or serial dilution of hits identified from the library screen in 0.5% DMSO for 4 hours. ONE-Glo™ EX reagent (Promega) was added to measure the luciferase activity using an EnVision plate reader. Relative luminescent units were plotted against corresponding drug concentrations and fitted with a standard four parameter sigmoidal curve with GraphPad Prism. EC_50_ values are shown for the three compounds that produced a dose response curve.

In order to expand on our initial pilot screen with the TESLR construct integrated into a stable cell-line,[28] we chose to screen the Selleckchem Bioactive Screening library (a collection of 2,035 small molecules), which includes FDA approved drugs, compounds that have entered clinical studies and validated tool compounds, see Fig 1. The TESLR construct is stably integrated into an engineered cell line and produces functional luciferase when three exons are skipped in an MDM2 based minigene construct.[28] This screen showed a low initial hit rate of 11 hits that had significant activity in replicates (see Fig 1 and Table 1), which is consistent with our previous experience with a pilot screen.[28]. The 11 initial hits were then tested in a dose-response format in the TESLR assay, which confirmed the activity of three compounds (see Fig 2).

**Fig 3:**
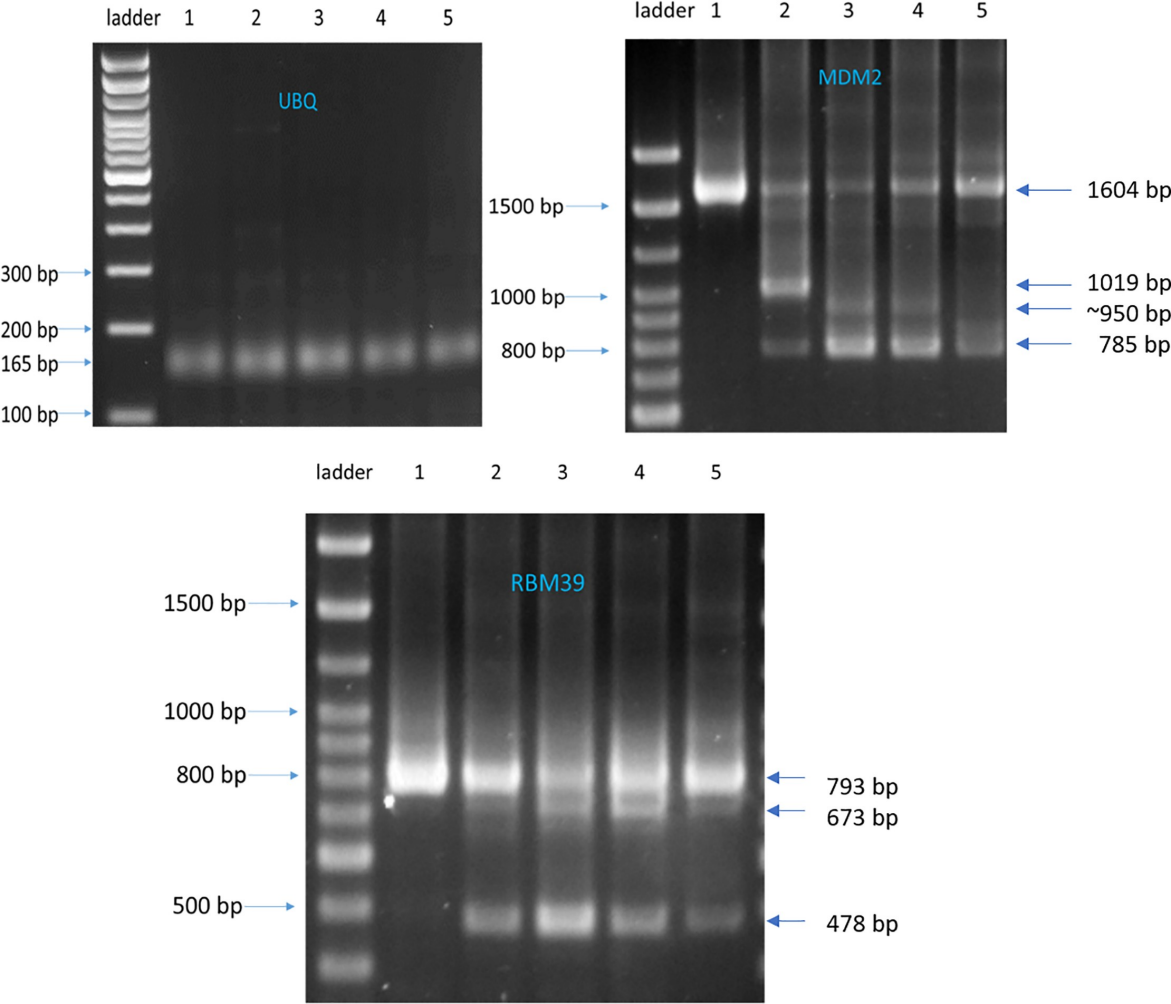
Effects of SD6, compound 1, 2, and 3 on pre-mRNA splicing for the ubiquitin, MDM2 and RBM39 genes in Rh18 using RT-PCR. AS patterns were identified with compound treatment for MDM2 and RBM39 transcripts. Ubiquitin (UBQ) transcripts were used as a control since these are not spliced. 100 bp DNA Ladder; (1) 0.5% DMSO; (2) SD6: 10 μM; (3) Compound **1**: 10 μM; (4) Compound **2**: 5 μM; (5) Compound **3**: 10 μM. Rh18 cells were treated with either DMSO control or compounds for 4 h. Total RNA was extracted and converted to cDNA. PCR was performed using corresponding primers and PCR products were subjected to 3% agarose gel electrophoresis.

The three confirmed hits were milciclib [30] (compound **1,** SRI-030867), PF-3758309 [31] (compound **2,** SRI-030868) and PF-562271 [32] (compound **3,** SRI-030871). These three actives were further confirmed by measuring MDM2 mRNA splicing modulation with RT-PCR using a previously reported gel-based assay that produced the expected MDM2 splicing alterations that are seen with SD6 treatment. Notably, the pattern of AS observed with compounds **1**, **2**, and **3** was not the same as that observed with SD6 (see Fig 3B).[19] A dominant AS isoform at size of 1019 bp observed with SD6 treatment was not apparent in the gel with compound **1**, **2**, or **3** treatment, while an AS isoform of 785 bp was dominant with compound **1** and **2** treatment, while another AS isoform of lesser abundance (~950 bp) appeared with compound **1** and **2** treatment. A similar MDM2 AS isoform was previously reported following sudemycin C1, D1 and E treatment [19, 20]. The MDM2 AS pattern resulting from compound **3** was different than that observed with all other compounds, exhibiting the apperance of a dominant MDM2 transcript (the same size as the DMSO control) and an AS isoform of 785 bp, as shown in Fig 3B.

To explore other AS splicing patterns caused by these compounds in additional genes, we chose RBM39 as a model, since it has previously been shown to undergo AS induced by SD6. [20] Interestingly, RBM39 is a member of the U2AF65 family of proteins that co-localizes in the nucleus with core spliceosome proteins and has been shown to play a role in both steroid hormone receptor-mediated transcription and alternative splicing.[34] Sudemycin induced splicing changes for RBM39 have been characterized and confirmed using RT-PCR.[20] Our results for compounds **1, 2** and **3** were consistent with the previously observed sudemycin induced splicing changes. Three differential RBM39 AS isoforms were identified with compound treatment, with a transcript at 673 bp showing the greatest abundance after treatment with compounds **1** and **2** (Fig 3C). In general, we concluded that AS patterns induced by compounds **1** and **2** were the similar but distinct from the sudemycin induced changes and that these changes are slightly different from the AS patterns resulting from compound **3** treatment. It should be pointed out that these experiments suggest that genes showing AS changes within cells exposed to **1**, **2**, or **3** have the potential be used as pharmacodynamic biomarkers for these kinases, as we have previously shown for the SF3B1 targeted agent SD6.[27, 35]

### The three hits are all kinase inhibitors that show potent biochemical and cell-based inhibition of the phosphorylation of substrates

**Fig 4:**
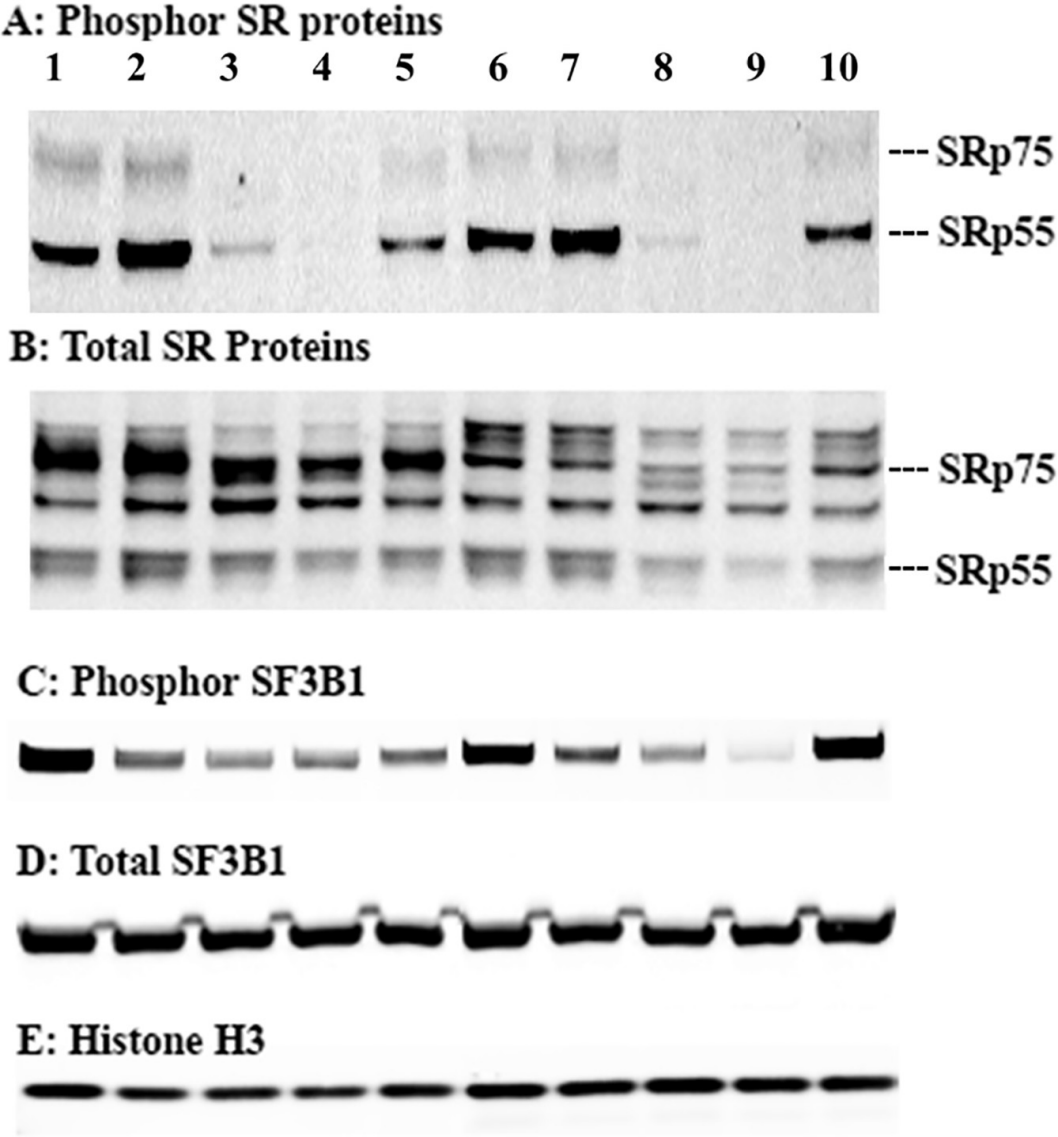
Effects of SD6, compound 1, 2, and 3 on phosphorylation and total protein of SF3B1 and SR proteins. Nuclear extracts blotted with antibodies to phosphor-SR, total SR, phosphor-SF3B1, total SF3B1 or histone H3 proteins from HCT-116 cells and Rh18 cells treated with DMSO, SD6 or compounds **1**, **2**, or **3** for 4 h. Lanes for all gels: Left to right: Lanes 1 to 5 are nuclear extracts from HCT116 cells. Lane 1: DMSO; Lane 2: 10 μM SD6; Lane 3: 10 μM compound **1**; Lane 4: 5 μM compound **2**; Lane 5: 10 μM compound **3**; Lanes 6 to 10 are nuclear extracts from Rh18 cells. Lane 6: DMSO, 4 h; Lane 7: 10 μM SD6; Lane 8: 10 μM compound **1**; Lane 9: 5 μM compound **2**; Lane 10: 10 μM compound **3**. These blots are representative of three independent experiments. Procedures can be found in the SI in the Materials and Methods section.

All three hits have published discovery-related selectivity data from different small kinase panels, however none of these panels included members of the CMCG kinase family, which are known to be involved in AS regulation.[30–32]. Our previous pilot screening results[28] together with the fact that these three confirmed hits were known to be kinase inhibitors(see Fig 4 for the chemical structures of these three hits), led us to suspect that these compounds target splicing regulatory kinases. Therefore, we screened these compounds against a panel of kinases known to be involved in the regulation of AS. We then determined the inhibitory IC_50_s for any compound that showed > 50% inhibition at 1 μM for any enzyme in the panel. As shown in Table 3, the confirmed hits showed potent biochemical inhibition of splicing regulatory kinases and other members of the CMCG family, which has not previously been reported for any of these drugs. The relevance of the biochemical data to the cell-based activity of these compounds was demonstrated by measuring total and phosphorylated (phosphor) forms of SR proteins by immunoblots, since these phosphorylation events are known to affect AS (see Fig 4A and 4B). Additionally, we investigated the effect of these drugs on SF3B1 phosphorylation (see Fig 4C and 4D), using 0.5% DMSO as a negative control, since a splicing modulatory natural product has been shown to reduce the phosphorylation of SF3B1.[36] Interestingly, compounds **1**, and compound **2** reduced phosphorylation of both SR and SF3B1 proteins significantly in HCT116 and Rh18 cell lines, while treatment SD6 did not inhibit the phosphorylation of SR proteins but did inhibit the phosphorylation of SF3B1, which is expected based on the results with pladienolide B in JeKo-1 cells.[36] Compound **3** treatment inhibited the phosphorylation of SR proteins in both HCT116 and Rh18 cells, but to a lesser extent than compound **1** or **2**. Compound **3** also inhibited the phosphorylation of SF3B1 protein in HCT116 cells, but not in Rh18 cells. SR proteins are known to be substrates for CLK and SRPK family members,[37] and DYRK1A has been reported to phosphorylate SF3B1[38]; however, the functional role of SF3B1 phosphorylation is not currently well understood. Based on the kinase inhibition profile of these compounds (Table 3) the reduction of SR protein phosphorylation may be attributed to inhibition of CLKs for compounds **1** and **2**, and the modest inhibition of CLKs by compound **3** may explain the lack of reduction of phosphor-SR protein in cells exposed to this compound. The marked reduction in phosphor SF3B1 by compound **3** points to inhibition of a hypothetical SR kinase that was not included in the panel shown in Table 3. Alternately, it is possible that FAK plays an unrecognized role in the phosphorylation of SF3B1. These results also imply a possible role of SF3B1 phosphorylation in AS, which could be the subject of further experiments.

**Table 3:** Biochemical kinase inhibition data of the confirmed TESLR hits with structures.

	Compound		
	1	2	3
Kinase	IC_50_ nM	IC_50_ nM	IC_50_ nM
AKT3	NA	54	NA
CDK2	45*	NA	203
CLK1	3	27	141
CLK2	1	6	83
CLK3	425	485	NA
CLK4	13	48	155
DYRK1A	17	NA	NA
DYRK1B	5	NA	NA
DYRK2	48	NA	NA
SRPK1	NA	170	NA
SRPK2	NA	NA	NA
SRPK3	NA	189	NA
CK2	NA	NA	NA
CK2a2	NA	NA	374
GSK3α(h)	NA	NA	221
GSK3β(h)	NA	NA	447
MAPK1	NA	NA	NA
MAPK2	NA	NA	863
PYK2	NA	NA	117
PAK4	NA	1.3*	396
FAK	NA	NA	115 (1.5)*

*Previously reported designated target data. NA: Not Active (less than 50% inhibition at 1 micromolar). The kinases NEK2, PIM1 and PIM2 were also investigated and found ‘not active’ for all three compounds examined. (See Table 1 for assay substrate information and additional details)

Focused screening of selective tool compounds and kinase inhibitory drugs uncovered additional active compounds in the TESLR screen that inhibit a subset of splicing regulatory kinases DYRK, CLK, or CK but not SRPK 1 or 2

**Fig 5:**
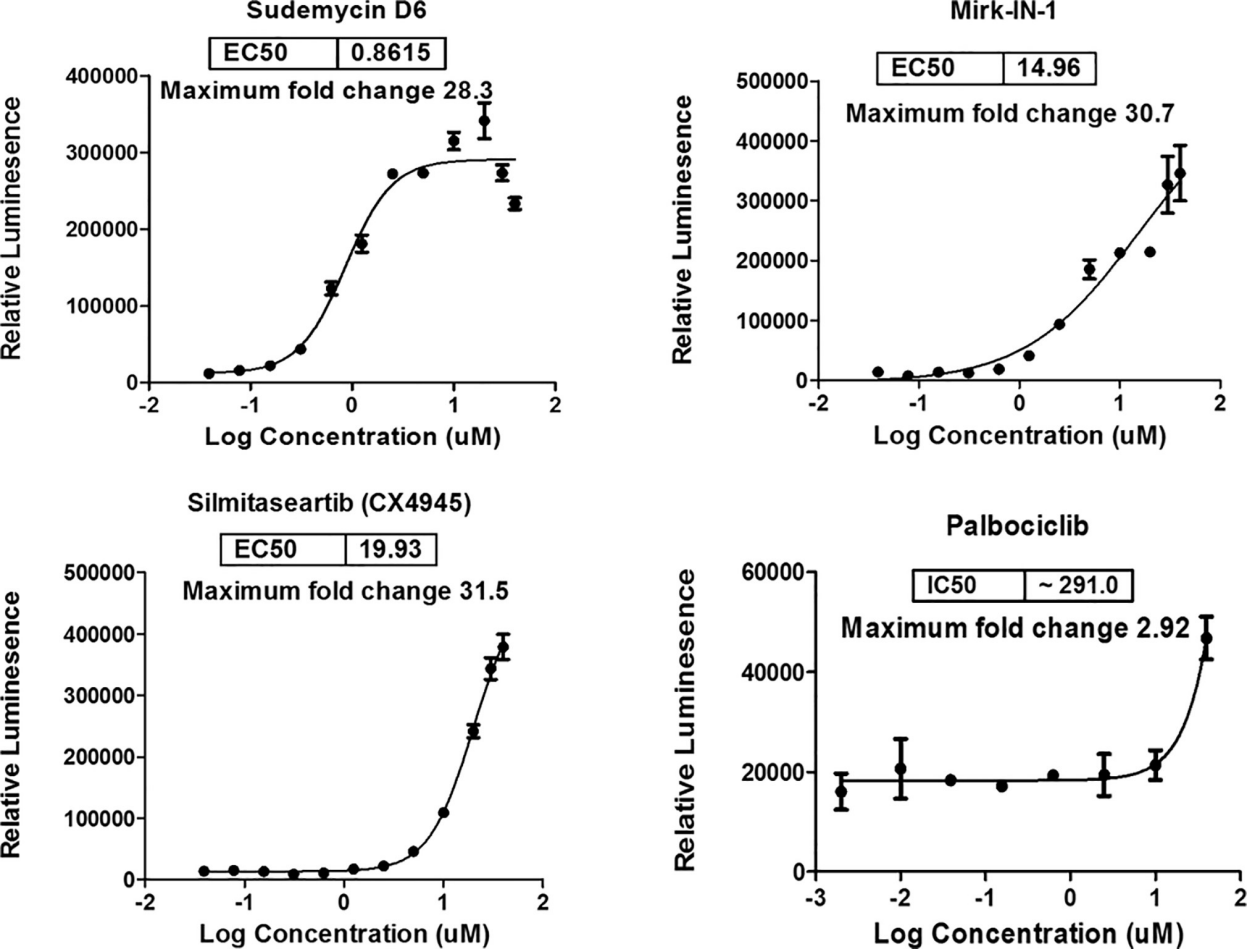
Tool compound dose response. SK-MEL-2/Luc-MDM2 stable cells were plated at a density of 20,000 cells/well in 96-well plates and incubated overnight at 37°C in 5% CO_2_. The following day, cells were treated with serial dilutions of the indicated compounds for 4 hours and ONE-Glo™ EX reagents (Promega) were added to measure the luciferase activity. Curves not shown for drugs with IC_50_s >> 10 μM.

Given the potent and previously unrecognized splicing kinase inhibitory activity of these drugs against this kinase panel, we also decided to investigate the activity of other known splicing kinase inhibitory drugs and tool compounds that were not included in the initial TESLR screen. To this end we performed a dose-response screen with Mirk-IN-1 (a known DYRK1A inhibitor),[39] Sphinx31 (a selective SRPK1 inhibitor)[40] dinaciclib (a FDA designated orphan drug and a potent CDK 1, 2, 5, and 9 inhibitor),[41] SRPIN340 (SRPK 1 and 2 inhibitor),[42] SNS-032 (CDK 2,7 and 9 inhibitor),[43] flavopiridol (Alvocidib, a FDA designated orphan drug and a potent pan-CDK inhibitor),[44] palbociclib (a non-selective CDK/multi-kinase inhibitor that is a FDA designated orphan drug),[45] and silmitasertib (a CK2 inhibitor in Phase II).[33] Of these compounds we found that only Mirk-IN-1, palbociclib and silmitasertib were active (IC_50_s in the range of 10–300 μM) in the TESLR assay, while the other splicing kinase inhibitors showed IC_50_s >> 10 μM in inducing triple exon skipping (Fig 5). This shows that the type of alternate splicing detected by the TESLR assay is not induced by SPRK inhibitors and also indicates that the inhibition of the DYRKs, casein kinase 2 (CK2), or CLKs 1, 2, and/or 4 (and the resulting inhibition of phosphorylation of SR proteins or SF3B1) were the likely causes of the triple-exon skipping observed in the TESLR screen with compounds **1**, **2**, **3**, and palbociclib, although the possibility that inhibition of FAK or other non-designated splicing kinases may also be involved, was not excluded by these studies. These results are completely consistent with recent findings from the chemoproteomic analysis of 243 kinase inhibitory drugs and tool compounds, which showed that drugs (e.g. palbociclib) and tool compounds that were touted as ‘selective’ actually potently inhibit numerous kinases, in addition to their designated targets.[45] Notably this comprehensive work clearly shows that palbociclib (trade name: Ibrance) has some affinity for CLK1 (IC_50_ = 276 nM),[45] which presumably accounts for the modest splicing modulation observed with this approved antitumor drug.

### Interrogating the SF3B1 small molecule binding site: The development of a competitive antagonist assay

**Fig 6:**
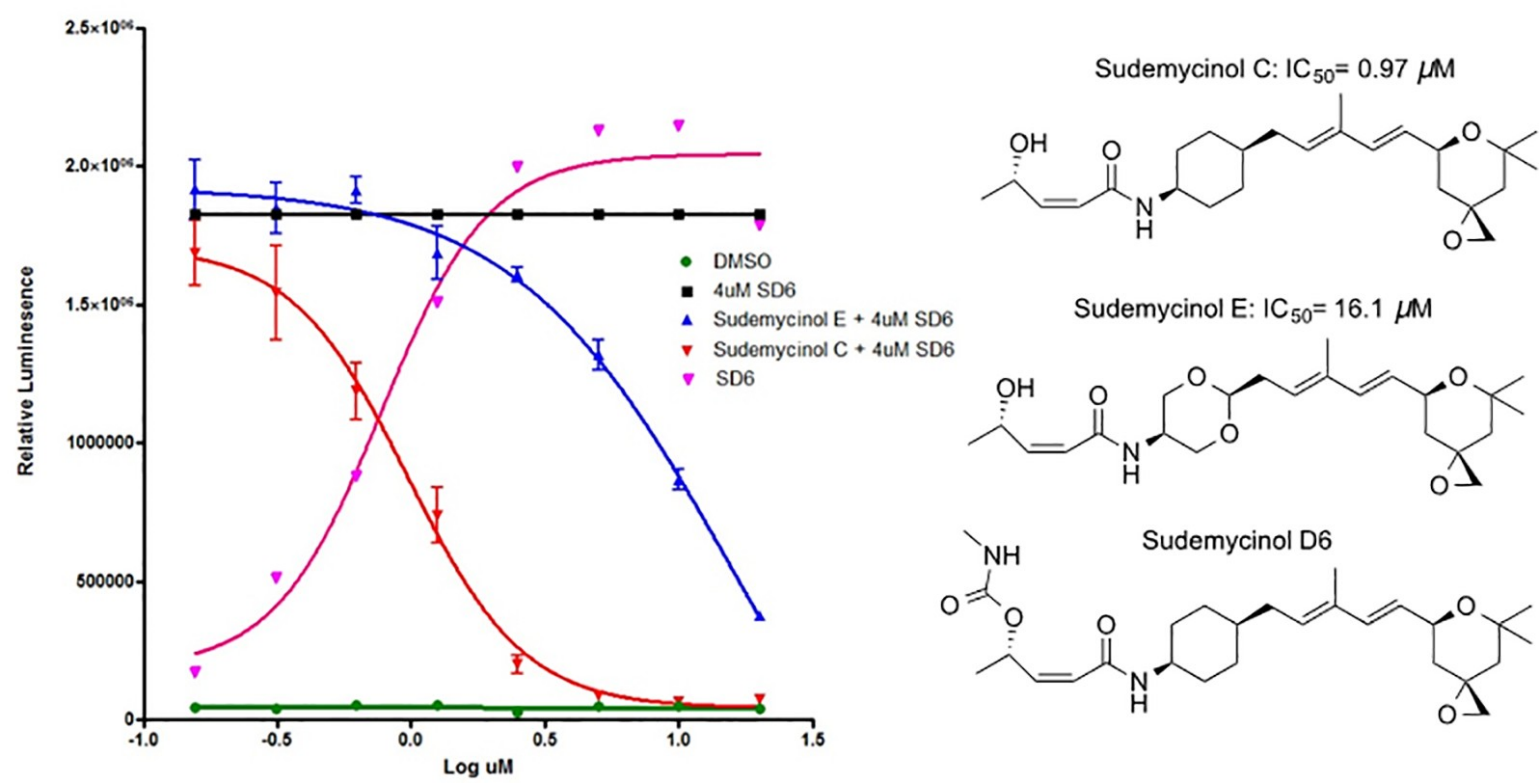
Competitive antagonist assay. (Left Panel) The assay that identified two antagonists of SD6 activity in the TESLR screen, which are useful for the determination of SF3B1 binding. Relative luminescent units were plotted against corresponding drug concentrations and fitted with a standard four parameter sigmoidal curve with GraphPad Prism. (Right Panel) The structures of the antagonists and SD6. See SI for chemistry details.

**Fig 7:**
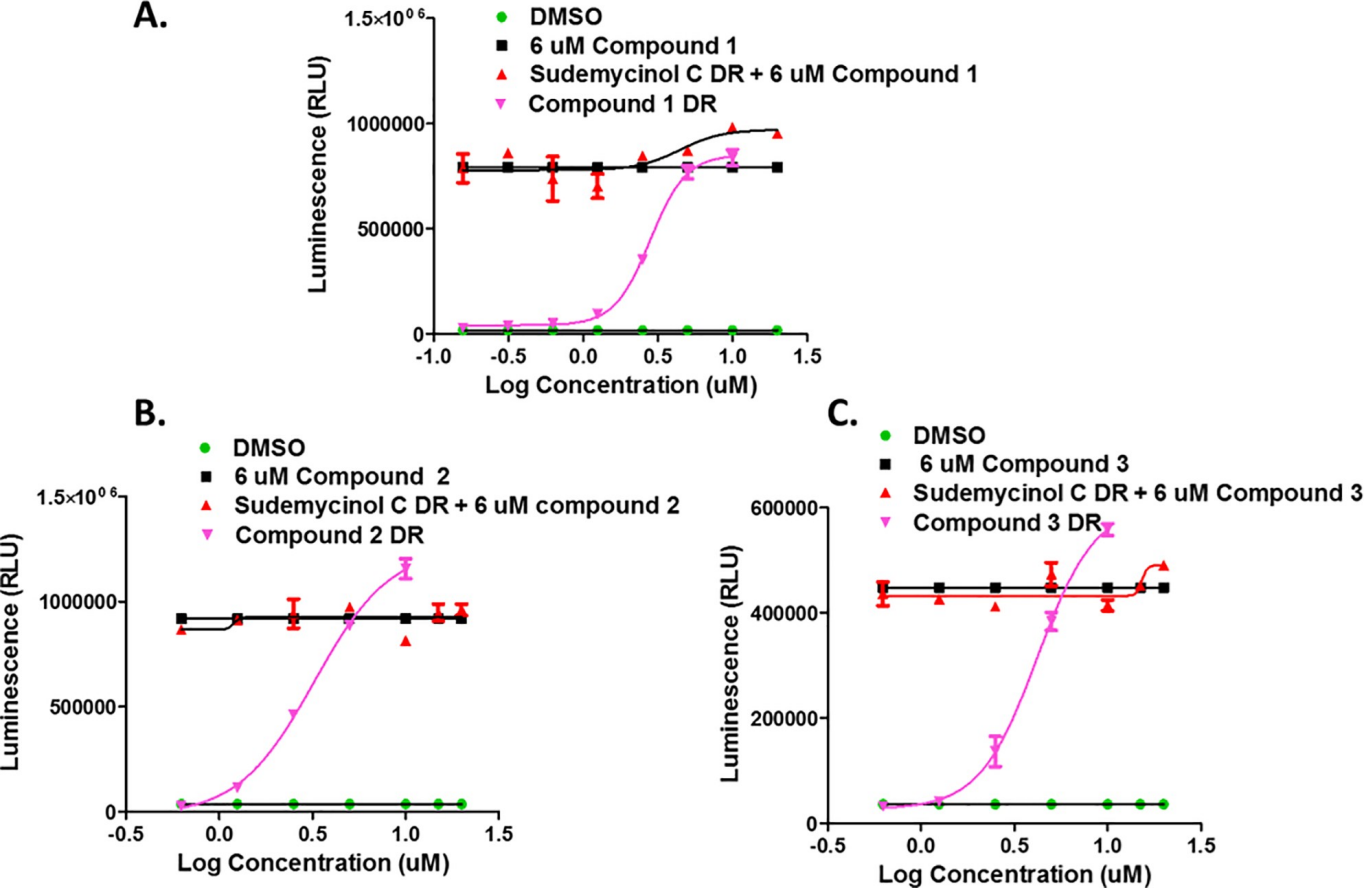
Effect of compound 1, 2 and 3 in the antagonist assay. SK-MEL-2/Luc-MDM2 stable cells were plated at a density of 20000/well in 96-well plates and incubated overnight at 37°C in 5% CO_2_. The following day, some cells were treated with 6 μM of compound **1**, **2** or **3** for one hour. After one-hour pre-treatment, serial dilutions of the sudemycinol C were added to the pre-treated cells for another 4 hours. At the same time, some cells were treated with 0.5% DMSO or serial dilutions of the compound **1**, **2** or **3** only, for 4 hours. At the end of incubation time, ONE-Glo™ EX reagents (Promega) were added to measure the luciferase activity. On the graph, value of DMSO and 6 μM of compound **1**, **2** or **3** were the average of the reading. A, B and C are the analyzed data corresponding to compound **1**, **2** and **3** single and combination treatment. DR: dose response.

In order to better understand the activity profile of TESLR hits we also decided to develop an improved assay for SF3B1 small molecule binding, since it is always possible that these hits could directly interact with SF3B1. We therefore decided to take advantage of the novel observation from the Jurica lab that ‘inactive’ analogs of natural product SF3B1 ligands can displace potent natural product splicing modulators in cell-free *in vitro* systems.[46] Since this report indicated that it was possible that our previously synthesized natural product analogs could be SF3B1 antagonists we screened a set of 14 of ‘inactive’ (minimal to no TESLR activity and non-cytotoxic at the concentrations investigated) sudemycin and herboxidiene analogs in the TESLR assay in the presence of moderate concentrations of SD6. This assay identified two compounds that were able to potently antagonize the activity of SD6 as shown in Fig 6. Using this assay format, we were then able to show that sudemycinol C did not antagonize the TESLR activity of compounds **1**, **2**, or **3**, which indicates that these hits do not bind to the same site on SF3B1 as SD6 (see Fig 7), which is consistent with our proposal that the inhibition of subsets of splicing regulatory kinases accounts for the splicing modulatory activity of these drugs. These results represent the first demonstration of SF3B1 antagonism in cells and the first HTS assay useful for the determination of binding to the splicing modulator binding site of SF3B1.

## Discussion

One major goal of this work was the discovery of new splicing modulatory drug-like lead compounds that exhibit similar splicing changes to those observed with the SF3B1 targeted natural products and analogs. To accomplish this, we developed the TESLR screen that was designed based on a prevalent, unusual and characteristic modification of pre-mRNA splicing we observed in tumor cells treated with SF3B1 antitumor natural products and analogs. We are therefore pleased to identify three post-Phase I antitumor drugs that may exhibit a substantial component of their activity through the modulation of splicing in combination with the activity for their designated target. It is interesting to note that though all available drugs and available clinical compounds were included in the screen, only antitumor drugs were identified, though this could simply be due to the fact that large number of kinase inhibitors are under clinical investigation and many investigational antitumor drugs are kinase inhibitors that typically exhibit polypharmacology.[45] Naturally, these results suggest that the repurposing of these multitargeted drugs should be envisioned in light of this new clinically-relevant MOA information. These results also clearly highlight the need for careful scholarship and scrutiny when using ‘selective’ drugs as tool compounds for experiments in cell biology.[47] Fortunately, an authoritative publication of inhibitor binding data for 243 clinical kinase inhibitors with 220 human kinases makes this important kinase selectivity data readily available to the biomedical science community.[45]

In order to evaluate the possible direct interaction of **1**, **2**, or **3** with the small molecule binding site of the SF3B subunit, we screened a small focused library of analogs of FR-901,464 and herboxidiene that show minimal activity in the TESLR assay, at the concentrations examined. Two of these compounds (sudemycinol C and E) were able to effectively reduce the TESLR activity of SD6 in a dose-dependent manner, presumably by competing with SD6 for the SF3B1 binding site without triggering the modulation of splicing detected by the TESLR assay. This observation is consistent with results from cell-free experiments, reported from the Jurica laboratory, which showed that binding to the small molecule SF3B1 site is necessary, but not sufficient, to induce splicing modulation and reported several different antagonists at this site.[46] Thus we discovered new simple antagonists and extended this type of assay into a format using living cells, which is useful for the high-throughput determination of SF3B1 small molecule binding. Using this screen, we were able to show that the activity seen with **1**, **2**, or **3** is not affected by the antagonist sudemycinol C, which is consistent with our hypothesis that these drugs exert their action through the inhibition of splicing modulatory kinases.

In summary, we report the novel characterization of three drugs as splicing modulators **1** (milciclib), **2** (PF-3758309), and **3** (PF-562271) that show potency on par with the SF3B1 targeted drug candidate SD6 in the TESLR screen, which reports on a triple-exon skipping event in MDM2 pre-mRNA that we first observed in tumor cells treated with sudemycin analogs.**[19]** We further demonstrated that in addition to modulation of splicing of the MDM2 gene these three drugs differentially induce AS in multiple genes that we examined, further supporting our hypothesis that the splicing modulatory activity is an important aspect of the antitumor activity of these three drugs. We also characterized the MOA of these drugs as the potent biochemical inhibition of a subset of the CMCG family of kinases including DYRK, CLK and CK. Additionally, we confirmed that these compounds show cell-based inhibition of the phosphorylation of SR proteins and SF3B1. Though the functional role of phosphorylation of SF3B1 is not currently fully understood,[38] our results suggest a possible regulatory role of this phosphorylation event on alternative splicing, and show that the SF3B1 protein appears to be a substrate for other kinases including the CLKs.

We also report the discovery of new potent SF3B1 antagonists (sudemycinol C and sudemycinol E) for the SF3B1-sudemycin binding site, and thereby confirm the previous report of the remarkable pharmacology of this critical spliceosome regulatory component.[46] We also show that these newly identified antagonists allow for a facile determination of the compounds that interact with the SF3B1 SD6 binding site, via a simple competition assay coupled to the TESLR splicing reporter screen. Using this new assay, we show that hit compound’s splicing modulatory activity in TESLR is not mediated through binding to the SF3B1 small molecule binding site, which is consistent with our hypothesis that they act through their potent inhibition of different splicing factor kinases. Our results also highlight a previously unanticipated portrait of the receptor-like pharmacology of SF3B1 splicing protein that is beginning to emerge.

These new insights suggest novel opportunities for the future clinical repositioning of these agents for additional indications in oncology, since these drugs have already been evaluated through Phase I or Phase II clinical studies. This improved understanding of the MOA of these clinical agents can lead to the effective translation of basic research results into better informed future clinical studies with these drugs, with an increased likelihood of therapeutic success. Specifically, our work suggests that the pre-clinical efficacy of compounds **1**, **2**, and **3** should be examined for possible clinical repurposing in MDS, CLL and AML. Our work also adds new tools compounds, and further clarifies the activity of known splicing modulatory compounds for basic research in cell biology. These interconnected outcomes support a growing recognition of the substantial potential of modulators of pre-mRNA splicing in cancer therapy, and also suggest a profile for a new chemotherapeutic class of multi-targeted splicing kinase inhibitors with other potential clinical applications, which is of interest to scientists across a broad range of disciplines from drug discovery through clinical practice in oncology.

## Supporting information

S1 File Synthetic procedures for analogs. (DOCX)

S1 Raw images (PDF)
