# Supplementary material for: An exon skipping screen identifies antitumor drugs that are potent modulators of pre-mRNA splicing, suggesting new therapeutic applications
Source: PLoS One. 2020 May 29;15(5):e0233672. doi: 10.1371/journal.pone.0233672 (PMC7259758; doi:10.1371/journal.pone.0233672)
Supplement: S1 File — (DOCX) [file pone.0233672.s001.docx]

Supporting Information

An exon skipping screen identifies antitumor drugs that are potent modulators of pre-mRNA splicing, suggesting new therapeutic applications

Yihui Shi,^1,3^ Walter Bray,^2^ Alexander J. Smith,^2^ Wei Zhou,^1^ Joy Calaoagan,^1^ Chandraiah Lagisetti,^1^ Lidia Sambucetti,^1^ Phillip Crews,^2^ R. Scott Lokey,^2^ Thomas R. Webb*^1,2,4^

**Synthetic Procedures for Natural Product Analogs**

Scheme S1. General scheme for the synthesis of herboxidiene analogs.

The following compounds have not been previously reported but were prepared based on our published work.[1-4]

**CLA-G-10:** CLA-G-10 is prepared from diol CLA-G-03B by treatment with 2,2-dimethoxy propane and catalytic toluene-4-sulfonic acid. ^1^H NMR (400 MHz, Chloroform-*d*) δ 6.17 (d, *J* = 15.7 Hz, 1H), 5.75 (d, *J* = 11.4 Hz, 1H), 5.59 - 5.33 (m, 3H), 4.31 (dd, *J* = 11.3, 6.7 Hz, 1H), 3.67 - 3.52 (m, 2H), 2.67 (d, *J* = 4.8 Hz, 3H), 1.99 (t, *J* = 7.1 Hz, 2H), 1.72 - 1.41 (m, 17H), 1.29 (d, *J* = 9.0 Hz, 12H), 1.22 - 1.18 (m, 3H), 1.15 - 1.11 (m, 3H); ^13^C NMR (101 MHz, CDCl_3_) δ 165.15, 157.04, 135.85, 133.84, 131.77, 127.33, 126.14, 109.93, 78.69, 75.51, 72.61, 69.18, 68.43, 45.33, 44.88, 41.51, 36.69, 32.57, 30.97, 29.59, 29.46, 27.66, 27.44, 27.40, 27.31, 27.21, 25.83, 24.38, 20.62, 12.46; MS (ESI) *m/z* 533 (M+1)^+^

**CLA_E_59:** ^1^H NMR (400 MHz, Chloroform-*d*) δ 5.99 (dt, *J* = 15.7, 0.9 Hz, 1H), 5.48 (dd, *J* = 15.7, 8.1 Hz, 1H), 5.30 (d, *J* = 7.5 Hz, 1H), 4.17 - 4.02 (m, 1H), 3.86 - 3.70 (m, 1H), 3.60 (s, 3H), 3.50 - 3.40 (m, 1H), 2.84 - 2.70 (m, 2H), 2.58 - 2.45 (m, 1H), 2.40 - 2.27 (m, 2H), 1.88 (d, *J* = 4.5 Hz, 1H), 1.84 - 1.77 (m, 1H), 1.68 – 1.61 (m, 1H), 1.74 - 1.68 (m, 4H), 1.62 - 1.51 (m, 4H), 1.38 - 1.15 (m, 3H), 1.02 – 0.96 (m, 3H), 0.87 – 0.81 (m, 6H); ^13^C NMR (101 MHz, CDCl_3_) δ 171.83, 135.92, 134.30, 133.36, 130.96, 74.95, 74.20, 73.92, 57.41, 57.33, 51.58, 41.48, 39.71, 35.42, 35.10, 33.77, 31.31, 30.80, 23.38, 21.21, 18.56, 17.40, 13.13; MS (ESI) *m/z* 395 (M+1)^+^

**CLA_E_72**: ^1^H NMR (400 MHz, Chloroform-*d*) δ 6.27 (ddt, *J* = 14.2, 10.9, 1.6 Hz, 1H), 6.01 (d, 14.2 Hz, 1H), 5.60 - 5.51 (m, 1H), 3.83 (ddd, *J* = 11.4, 7.6, 5.7 Hz, 1H), 3.73 (d, *J* = 11.0 Hz, 1H), 3.68 (s, 1H), 3.50 (d, *J* = 10.0 Hz, 1H), 2.93 - 2.76 (m, 2H), 2.64 - 2.56 (m, 1H), 2.50 - 2.39 (m, 2H), 1.95 (s, 1H), 1.89 (dt, *J* = 12.9, 2.9 Hz, 1H), 1.81 - 1.71 (m, 7H), 1.70 - 1.60 (m, 6H), 1.59 - 1.50 (m, 4H), 1.41 - 1.14 (m, 7H), 1.10 - 1.05 (m, 3H), 1.04 - 0.95 (m, 2H); ^13^C NMR (101 MHz, CDCl_3_) δ 171.85, 138.97, 136.92, 125.27, 124.13, 82.09, 74.44, 73.38, 57.37, 57.35, 51.56, 43.76, 41.51, 39.70, 35.49, 35.30, 31.06, 29.61, 29.01, 27.93, 26.49, 26.23, 26.10, 23.55, 21.12, 13.60; MS (ESI) *m/z* 449 (M+1)^+^

**CLA_E_91:** ^1^H NMR (400 MHz, Chloroform-*d*) δ 6.22 (dd, *J* = 15.3.0, 10.9 Hz, 1H), 5.85 (dd, *J* = 11.0, 1.6 Hz, 1H), 5.51 (ddd, *J* = 15.3, 8.4, 2.8 Hz, 1H), 3.81 - 3.71 (m, 1H), 3.67 (s, 3H), 3.52 (d, *J* = 10.7 Hz, 2H), 2.92 - 2.78 (m, 2H), 2.68 - 2.58 (m, 1H), 2.46 (ddd, *J* = 14.9, 6.8, 4.8 Hz, 2H), 1.98 (t, *J* = 4.8 Hz, 1H), 1.85 – 1.71 (m, 8H), 1.70 - 1.59 (m, 3H), 1.56 - 1.45 (m, 6H), 1.36 -1.29 (m, 1H), 1.25 - 1.12 (m, 3H), 1.07 (d, *J* = 6.8 Hz, 3H), 1.04 - 0.92 (m, 2H), 0.88 (s, 3H), 0.82 (s, 3H); ^13^C NMR (101 MHz, CDCl_3_) δ 171.84, 138.52, 134.73, 127.34, 125.13, 89.99, 74.62, 73.36, 57.47, 57.43, 51.54, 43.78, 41.35, 39.71, 39.60, 35.44, 35.30, 33.84, 29.00, 28.16, 28.01, 27.96, 26.48, 26.24, 26.10, 21.16, 20.42, 15.01; MS (ESI) *m/z* 477 (M+1)^+^

**Reference**

1. Lagisetti C, Pourpak A, Jiang Q, Cui X, Goronga T, Morris SW, et al. Antitumor compounds based on a natural product consensus pharmacophore. Journal of medicinal chemistry. 2008;51(19):6220-4.

2. Lagisetti C, Pourpak A, Goronga T, Jiang Q, Cui X, Hyle J, et al. Synthetic mRNA splicing modulator compounds with in vivo antitumor activity. Journal of medicinal chemistry. 2009;52(22):6979-90.

3. Lagisetti C, Palacios G, Goronga T, Freeman B, Caufield W, Webb TR. Optimization of antitumor modulators of pre-mRNA splicing. Journal of medicinal chemistry. 2013;56(24):10033-44.

4. Lagisetti C, Yermolina MV, Sharma LK, Palacios G, Prigaro BJ, Webb TR. Pre-mRNA splicing-modulatory pharmacophores: the total synthesis of herboxidiene, a pladienolide-herboxidiene hybrid analog and related derivatives. ACS chemical biology. 2014;9(3):643-8.
