# Supplementary figures and images for: An exon skipping screen identifies antitumor drugs that are potent modulators of pre-mRNA splicing, suggesting new therapeutic applications
Source: PLoS One. 2020 May 29;15(5):e0233672. doi: 10.1371/journal.pone.0233672 (PMC7259758; doi:10.1371/journal.pone.0233672)

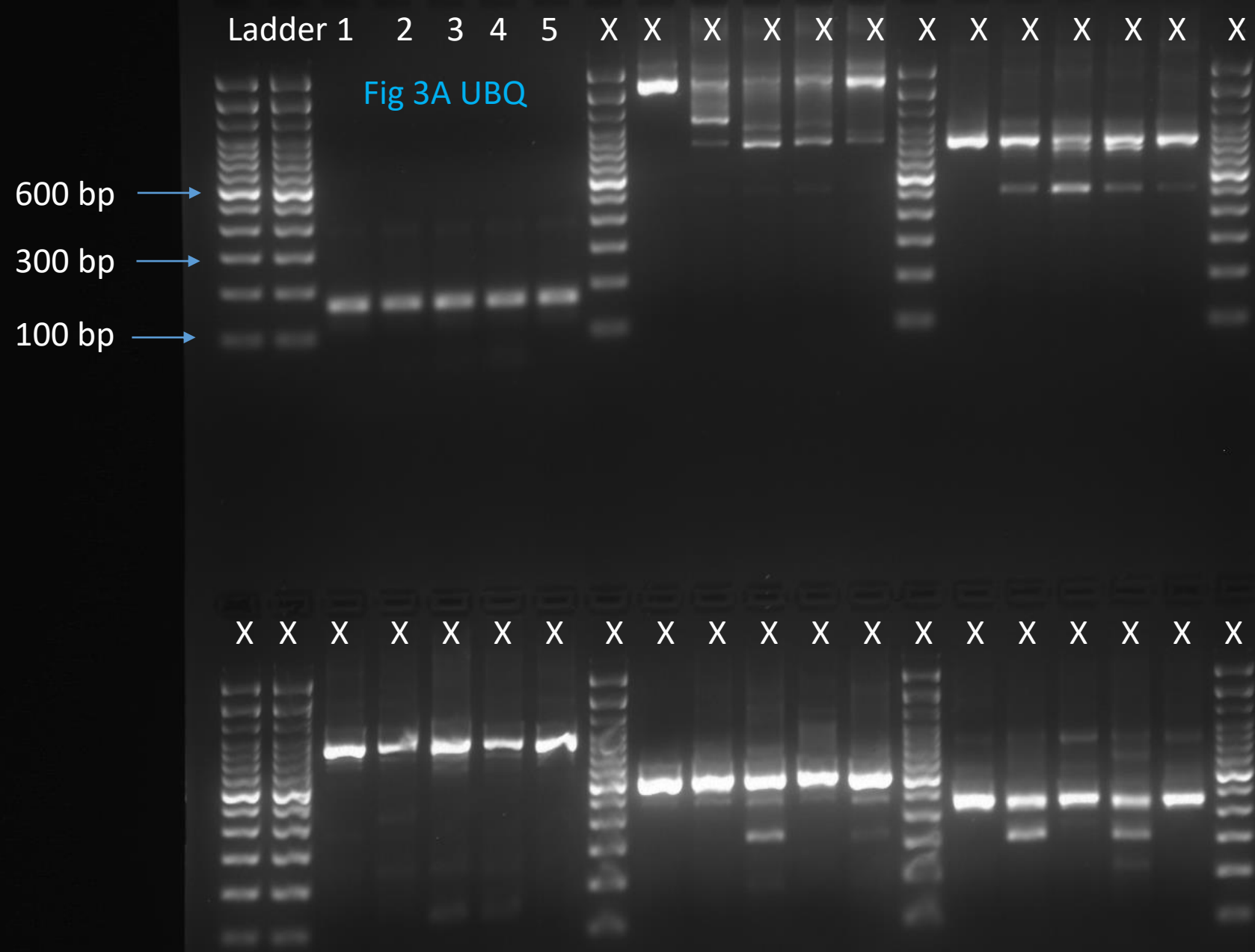

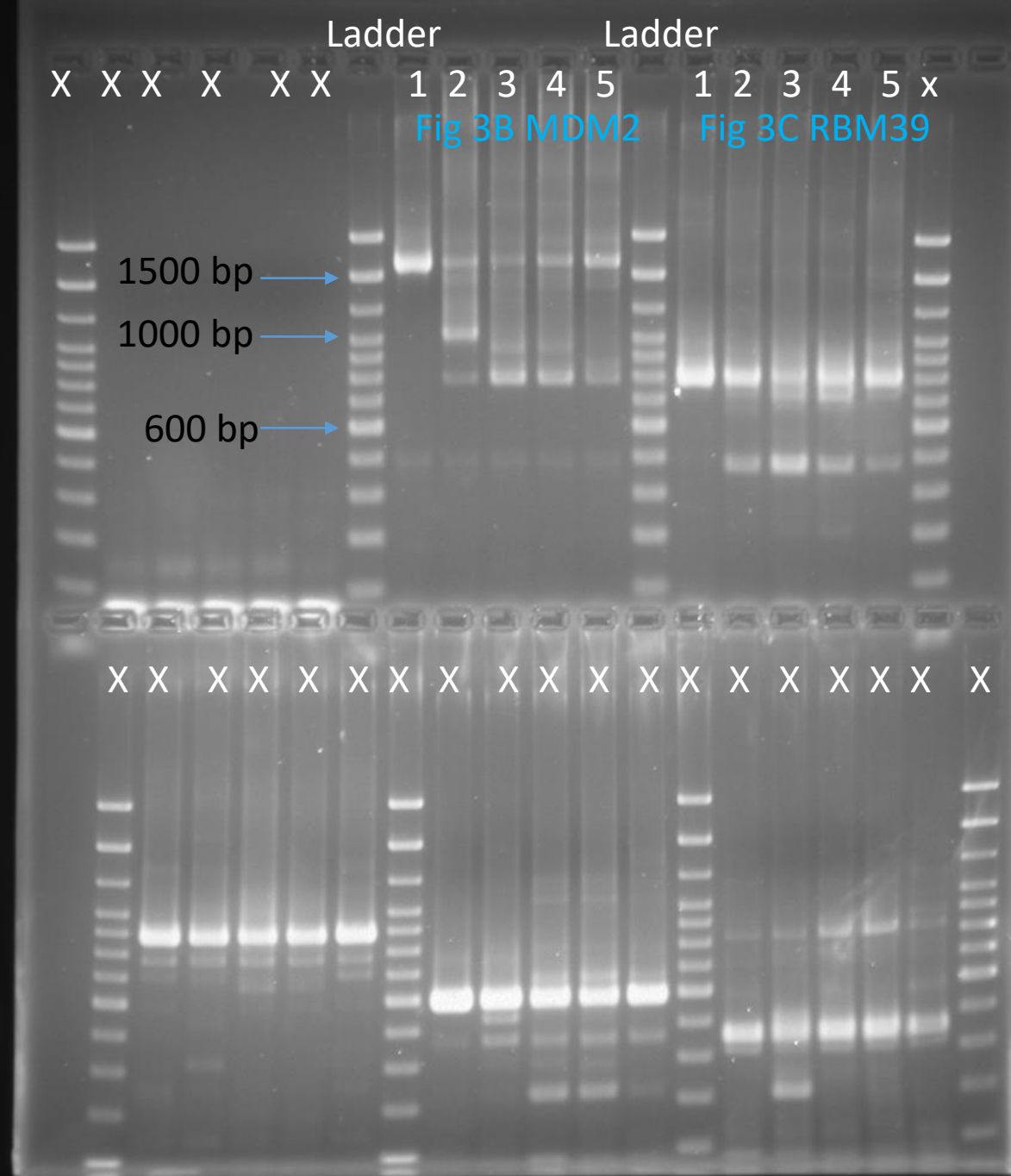

Fig. 4A Phosphor SR proteins

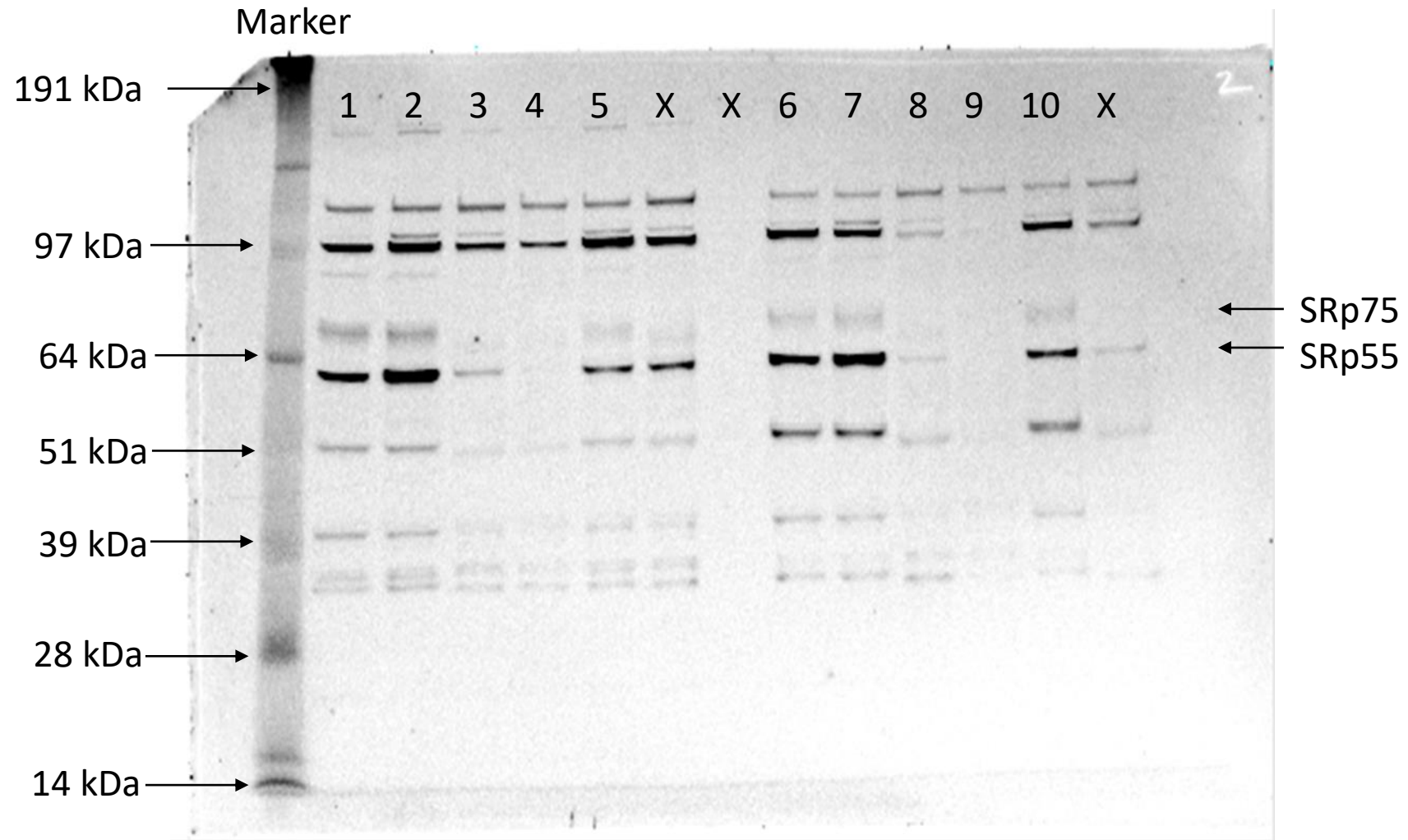

## Marker

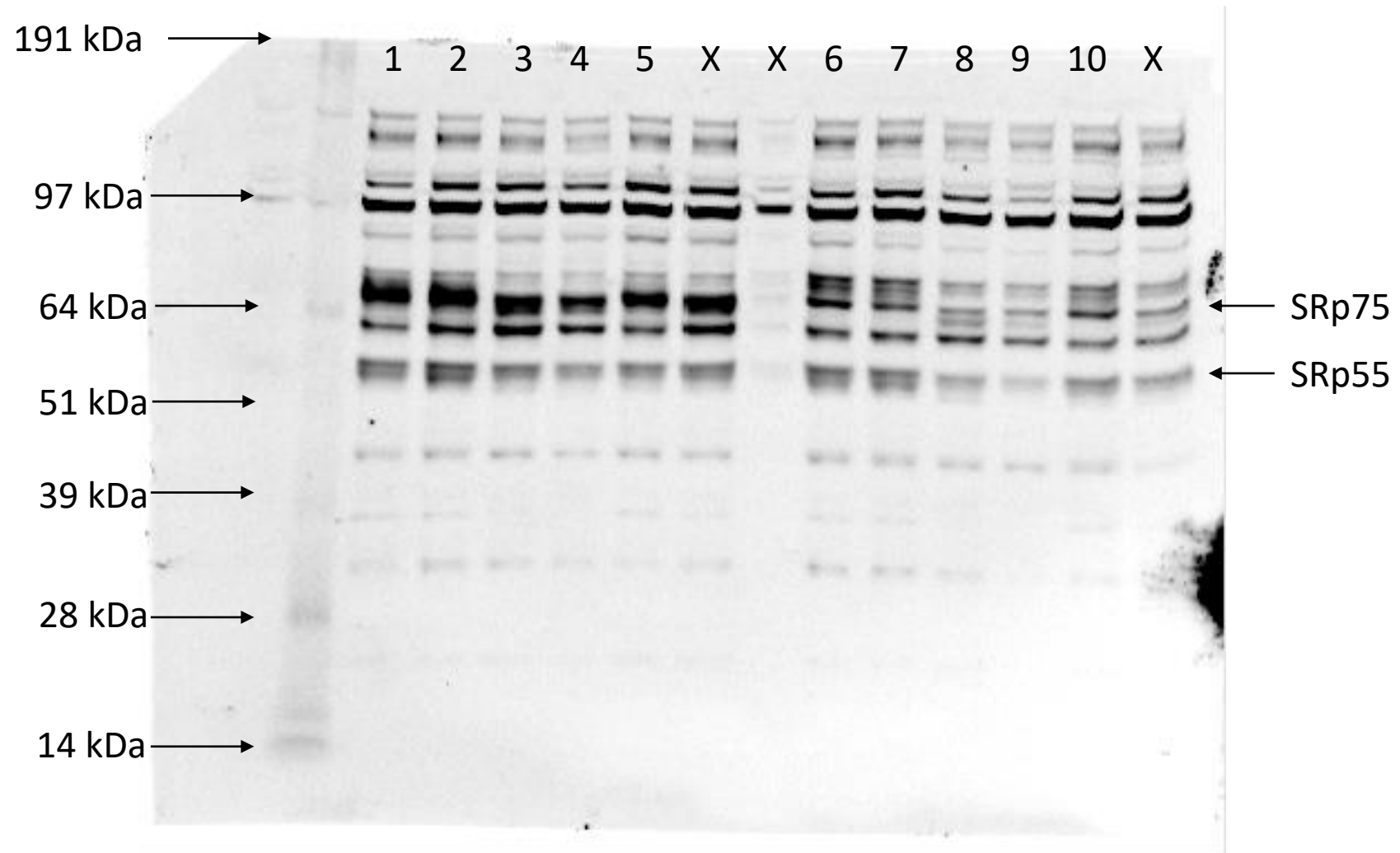

Fig. 4C Phosphor SF3B1

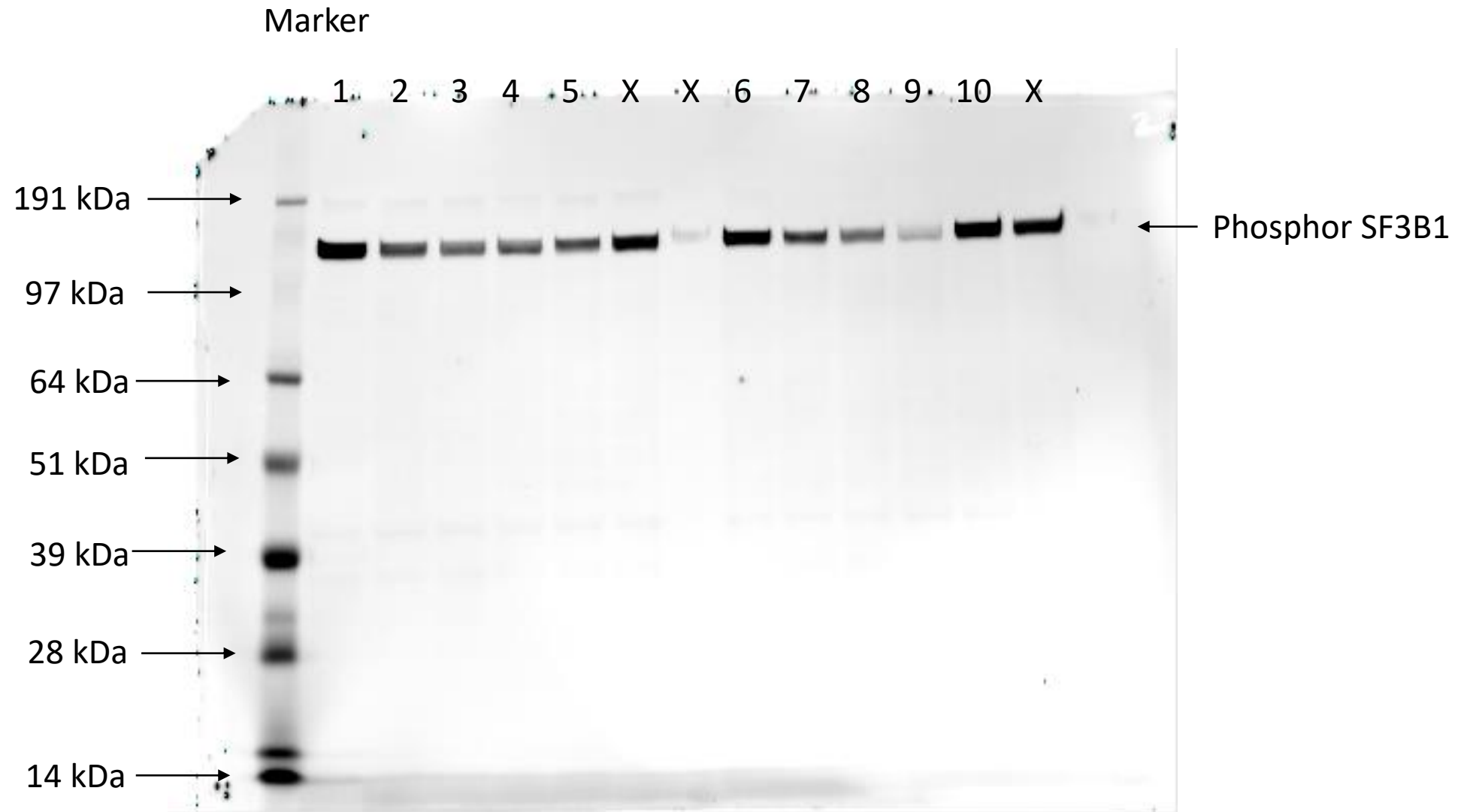

## Marker

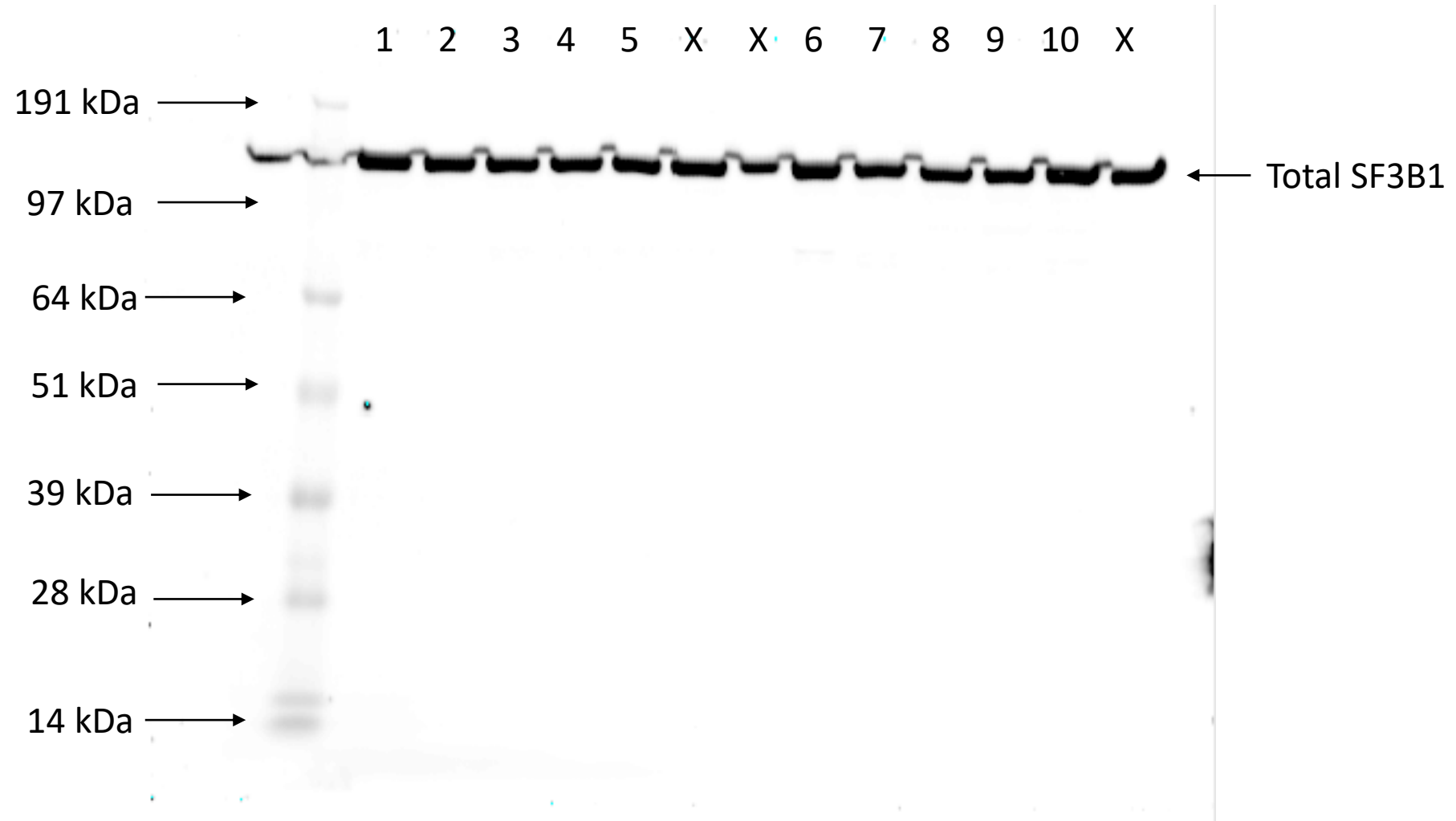

Fig. 4E Histone H3 and Lamin B1 (X)

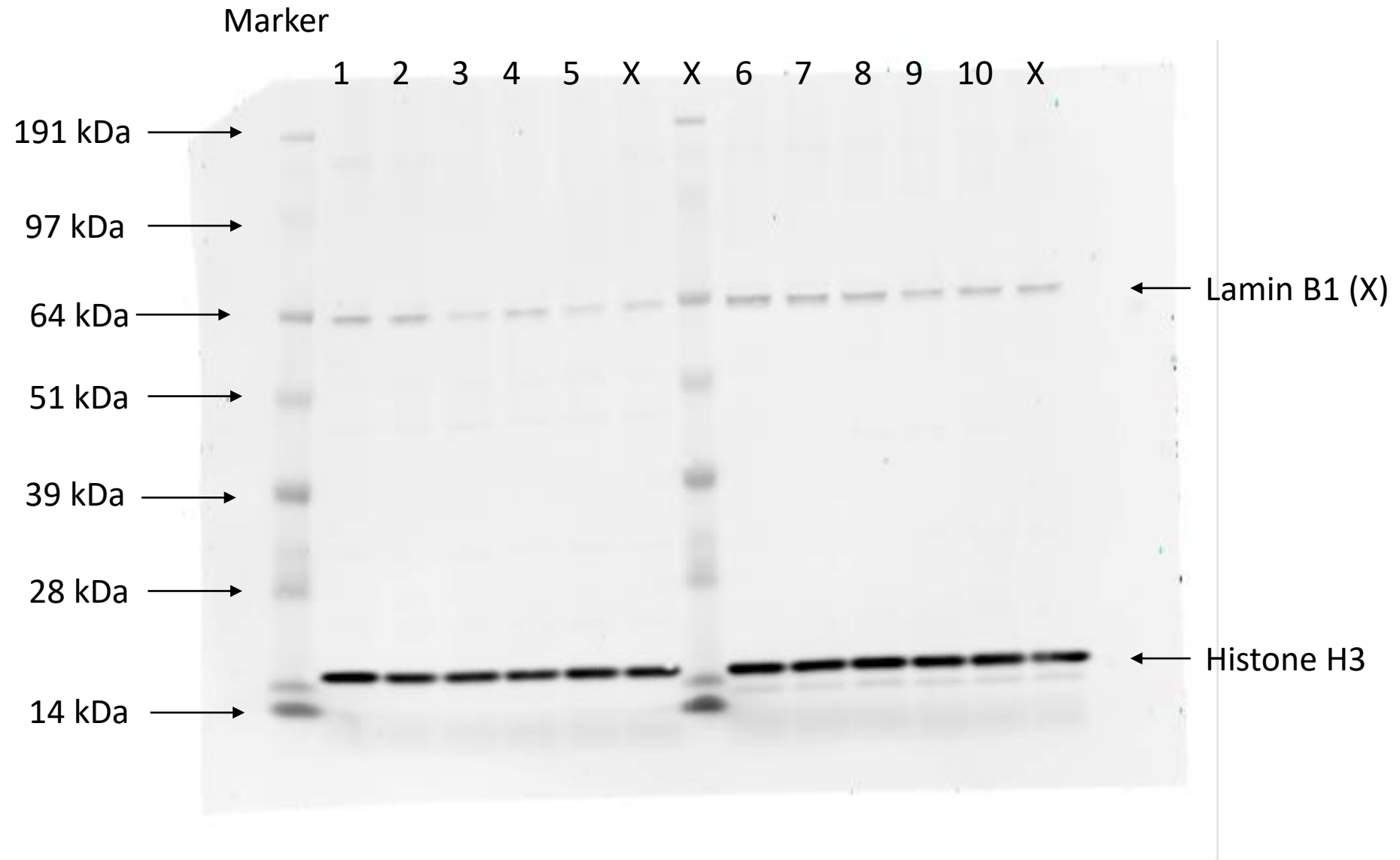

Supplement: S1 Raw images — (PDF) [file pone.0233672.s002.pdf]
